# Centric relation: A matter of form and substance

**DOI:** 10.1111/joor.13329

**Published:** 2022-04-20

**Authors:** Cinzia Fornai, Ian Tester, Kim Parlett, Cristian Basili, Helder Nunes Costa

**Affiliations:** ^1^ Vienna School of Interdisciplinary Dentistry (VieSID) Klosterneuburg Austria; ^2^ Institute of Evolutionary Medicine University of Zurich Zurich Switzerland; ^3^ Department of Evolutionary Anthropology University of Vienna Vienna Austria; ^4^ Faculty of Odontology University of Valparaíso Valparaíso Chile; ^5^ Department of Orthodontics Egas Moniz University (IUEM) Caparica Portugal; ^6^ Interdisciplinary Research Center of Egas Moniz (CiiEM) Caparica Portugal

**Keywords:** functional diagnostic, iatrogenic effects, occlusion, orthodontics, reference position, temporomandibular joint

## Abstract

The recent review article by Zonnenberg, Türp and Greene ‘Centric relation critically revisited – What are the clinical implications’? opens an important debate by addressing topics of central relevance in Dentistry, namely the relationship between occlusion and the condyle‐to‐glenoid‐fossa position, and the need for diagnostic assessment and therapeutic alteration of the condylar position in orthodontic patients. Zonnenberg, Türp and Greene concluded that the mandibular condyle is correctly situated in most orthodontic patients. Thus, in their view, orthodontists can disregard this aspect during treatment, and rely on the plastic properties of the masticatory supporting structures, while aiming at finishing the cases in a good occlusal relationship.

We think that this approach fails to consider that biological variation of the stomatognathic structures can also be pathological and that, as dental occlusion determines condylar relative position within the glenoid fossa, changes in the occlusion are likely to alter the original condylar‐to‐glenoid‐fossa relation. Hence, we claim that whenever the occlusal relationship must be changed, the clinician should carefully monitor the condyle position and the mandibular function to prevent possible iatrogenic effects.

To advance the discourse on the topic, we invite Zonnenberg, Türp and Greene to clarify their definition of ‘average patient’ and their interpretation of ‘full‐mouth orthodontic and orthognathic treatment’, their understanding of ‘biologically acceptable condylar relationship’, their justification of maximum intercuspation as reference position, the extent to which they think it is safe to rely on the TMJ resilience, and finally their alternative to centric relation in the treatment of patients needing condylar repositioning.

Zonnenberg, Türp and Greene^1^ objected to the application of the concept of centric relation in the treatment of the majority of orthodontic patients based on ‘semantic, conceptual and practical reasons’ (p. 1053). After carefully reading this contribution, we found ourselves in agreement with these authors on the lack of clarity surrounding the definition of centric relation, and the controversy over the methods used to locate this position. However, we think that this article is further evidence that the topic of therapeutic position is still gravely misunderstood, which provides us with the opportunity to elucidate some crucial points.

Based on the problems and limitations of the current definition of centric relation, Zonnenberg, Türp and Greene^1^ concluded that ‘If, however, the dentist accepts an existing MIP [maximum intercuspation]‐determined jaw relationship as being biologically acceptable for the vast majority of healthy dentate patients, there would be no need to conduct such assessments [of condylar position] as a part of routine examinations of the stomatognathic system’ (p. 1053). A patent confusion between what is biological (namely, occurring in nature, or phenotypically expressed in human beings) and what is clinically normal is apparent in this statement. Advancements in food technology in modern societies have decreased the selective pressure on the human masticatory system resulting in a high degree of morphological variation of the orofacial structures, or malocclusion.^2^ This fact must be acknowledged, but whether a condition is clinically acceptable should be determined by the practitioner based on an accurate and thorough assessment of the patient's signs and symptoms. Suggesting that the presenting condylar position in dentate patients is unquestionably correct because it is biologically determined is dismissive of the fact that biological variation (of the orofacial structures) can be pathological. On the other hand, it should not be assumed that a conscientious dentist would administer unnecessary diagnostic procedures to ‘healthy dentate patients’.

Zonnenberg, Türp and Greene^1^ continued along the same line (p. 1053): ‘… we should acknowledge that the average person will have a stable, repeatable and functional MIP [maximum intercuspation] that determines where the condyles and discs are located on their articular eminences. Therefore, no special assessment of the mandibular position needs to be carried out in these subjects’. The vagueness of the expression ‘average person’ might lead clinicians to the wrong understanding that it is safe to operate under the assumption that condylar position is functionally correct in most people, thereby neglecting a proper diagnosis in these patients. In fact, they acknowledged that ‘these so‐called discrepancies [namely the sliding between CR and MIP] are found within the vast majority of the normal population, which strongly suggests that they are a normal feature of intermaxillary relationships’. This statement emphasises the high variation of the occlusal relationship found in modern humans. Despite occlusal deviations having become ‘normal’ (i.e. frequent) in urbanised societies, it cannot be stated that, consequently, they are also clinically or functionally normal. Since Zonnenberg, Türp and Greene^1^ did not support their statement with relevant references, we refer to Pullinger, Seligman and Gornbein^3^ who showed that indeed a cut‐off value for sagittal occlusal slide length can be found, corresponding to 2 mm, based on their sample. In fact, none of their asymptomatic subjects had a slide longer than 2 mm, while only 6% of them had slide length between 1 and 2 mm.

Zonnenberg, Türp and Greene^1^ described three categories of patients in which condylar relative position should be assessed for the establishment of a ‘new jaw relationship*’* (p. 1054):

1. Edentulous (or partially edentulous) patients who require construction of partial or full removable denture prostheses.

2. Patients who need full‐mouth reconstruction, with or without implants.

3. Patients who need full‐mouth orthodontic and/or orthognathic therapy.

Two observations follow with regard to their point 3. First, it is unclear to us which patients the authors include within this category, but, if our understanding is correct, these subjects are those requiring a change of the occlusal relationship. In this case, the majority of the orthodontic patients might fall into this category. Second, any orthodontic intervention might have an influence on the stomatognathic system potentially affecting the temporomandibular joint (TMJ) configuration and, thus, its function. Therefore, a thorough functional diagnosis can alleviate unwanted iatrogenic events. In fact, as the authors' main claim is that MIP determines condylar position within the glenoid fossa, it is logical to assume that changes in the occlusion can potentially alter the original position of the condyles. Hence, if it is clinically established that the position of the condyles is functionally correct in a certain patient, a considerate practitioner should make sure that this spatial relationship is not altered through time by orthodontic or prosthodontic interventions. In this perspective, clinical and functional assessments of the stomatognathic system are crucial for monitoring the course of the treatment for the safety of patients and within the principles of good medical practice. Instead, Zonnenberg, Türp and Greene^1^ suggested that clinicians should rely on the capacity of the patients' TMJ structures to adapt through remodelling, which, we wish to remind, is reduced in case of TMJ inflammation or degenerative disorders, and is certainly more limited in adults than in children. It is also unclear what the effects of such a therapeutic approach might be on children's growth and health of the stomatognathic system in the medium and long terms.

On p. 1053, it is reported that The term “CR” is conceptually flawed because it is based upon the assumption that there is a place where condyles “should be”. While the position of the condyles within the fossae varies greatly in modern humans and changes through the lifespan of an individual, it has been demonstrated that TMJ skeletal morphology is associated with disc derangement dysfunctions.^4^ Thus, the relative position of the condyles within the glenoid fossa has functional implications, and it also seems that small changes in this relationship deriving from occlusal treatment result in an improvement of the symptoms in patients with temporo‐mandibular disorders.^5^ Accordingly, it is reasonable to assume that changes of the condylar position might also negatively affect the health of the masticatory organ.

In summary, to bring clarity into the debate, Zonnenberg, Türp and Greene^1^ are invited to clarify:

1. What they mean with average patient and which morphological, functional, diagnostic, demographic, criteria they use to define the average orthodontic patient.
2. Their interpretation of full‐mouth orthodontic and orthognathic treatment, namely which therapeutic goal these are intended to reach.
3. Their understanding of what is biologically acceptable in clinical settings, both in general and with respect to the relationship between occlusion and TMJ.
4. How they think MIP can define or allow to infer the condylar position within the glenoid fossa in orthodontic patients.
5. How MIP can be used as a diagnostic reference if the goal of an orthodontic treatment is to change the occlusal relationship.
6. Based on which clinical or biomechanical evidence can they suggest that it is safe to administer orthodontic treatment relying on the resilience and adaptability of the stomatognathic system rather than on comprehensive diagnostic and functional examination.
7. Additionally, they should explain their ‘proposals for a new perspective regarding how the condyle and disc should be related to the skull’ (p. 1052) in the patients presenting with ‘mandibular instability’.

In our view, association between morphology and function should be assessed comprehensively and investigated in relation to the occurrence and severity of symptoms. For this purpose, several monitoring approaches have been shown to be useful, including diagnostic imaging, jaw tracking systems^6^ and electromyograms. The use of accurate terminology is fundamental for beginning an expert debate on such a complex subject. The definition of centric relation in The Glossary of Prosthodontic Terms‐9^7^ was published after extensive debate and received a ‘consensus’ of only 29% among experts responsible for this definition. It is evident that crucial points must be addressed before this or other more suitable terms could be successfully used for diagnostic and research purposes. Hence, we agree with Zonnenberg, Türp and Greene that the term centric relation should be abandoned, and relaunch the use of Reference Position (RP) both for diagnostic and therapeutic purposes, namely an unstrained retral border position sensu Piehslinger, Celar, Celar, Jäger and Slavicek^8^ (see also ref. 9), which is obtained without exploiting the shock‐absorbing properties of the retrodiscal tissues. Differently from centric relation, the term Reference Position is advantageous because it is not evocative of a predetermined configuration of the condyle within the glenoid fossa. As explained in reference^8^ (p. 69), when Reference Position is achieved: ‘The mandible is in physiologic retral border position. All structures of the joint are unloaded, that is, the ligaments are not in tension in any direction. There is only minimum muscle activity and no pressure on cartilaginous structures’. In fact, Gerber^10^ had already warned against forcing a retruded position of the mandible because of iatrogenic risks. Finally, the high reproducibility demonstrated for Reference Position^8^ is further evidence of the clinical validity of this approach.

The advancement of Oral Medicine for the understanding of orofacial function and the improvement of diagnostics and treatments depends on all stakeholders' ability to sustain an honest and evidence‐based scientific discourse. Therefore, we are grateful to the Journal of Oral Rehabilitation and its Editor for hosting this open debate.

## CONFLICT OF INTEREST

The authors declare no conflicts of interest.

## AUTHORS' CONTRIBUTIONS

CF, IT and KP made substantial contributions to the conception of the manuscript. All authors made substantial contributions to the design of the manuscript. CF wrote the paper and all authors critically revised it. All authors approved of the version to be published and agreed to be accountable for all aspects of the work.

### PEER REVIEW

The peer review history for this article is available at https://publons.com/publon/10.1111/joor.13329.

## Data Availability

Data sharing is not applicable to this article, as no new data were created or analysed in this study.

## References

[1] Zonnenberg AJJ , Türp JC , Greene CS . Centric relation critically revisited—what are the clinical implications? J Oral Rehabil. 2021;48(9):1050‐1055. doi:10.1111/joor.13215 34164832

[2] Corruccini RS . How Anthropology Informs the Orthodontic Diagnosis of Malocclusion's Causes. The Edwin Mellen Press Ltd; 1999. ISBN:0‐7734‐7980‐5; 978‐0‐7734‐7980‐7.

[3] Pullinger AG , Seligman DA , Gornbein JA . A multiple logistic regression analysis of the risk and relative odds of temporomandibular disorders as a function of common occlusal features. J Dent Res. 1993;72(6):968‐979. doi:10.1177/00220345930720061301 8496480

[4] Pullinger AG , Solberg WK , Hollender L , Petersson A . Relationship of mandibular condylar position to dental occlusion factors in an asymptomatic population. Am J Orthod Dentofacial Orthop. 1987;91(3):200‐206. doi:10.1016/0889-5406(87)90447-1 3469906

[5] Ramachandran A , Jose R , Tunkiwala A , et al. Effect of deprogramming splint and occlusal equilibration on condylar position of TMD patients – a CBCT assessment. CRANIO®. 2021;39(4):294‐302. doi:10.1080/08869634.2019.1650216 31451061

[6] Kobs G , Bernhardt O , Kocher T , Meyer G . Accuracy of traditional clinical examination in combination with 3‐D computerized axiography for diagnosing anterior disk displacement with reduction. Stomatologija. 2004;6(3):91‐93.

[7] The glossary of prosthodontic terms: ninth edition. J Prosthet Dent. 2017;117(5s):e1‐e105. doi:10.1016/j.prosdent.2016.12.001 28418832

[8] Piehslinger E , Celar A , Celar R , Jäger W , Slavicek R . Reproducibility of the condylar reference position. J Orofac Pain. 1993;7(1):68‐75. 8467299

[9] Slavicek R . The Masticatory Organ: Functions and Dysfunctions. GAMMA Medizinisch‐wissenschaftliche Fortbildung‐AG; 2002.

[10] Gerber A . Kiefergelenk und Zahnokklusion. Deutsche Zahnärztliche Zeitschrift. 1971;26:119‐141. 5278940

